# Bovine Endometrium Drives and Responds to Divergence of In Vitro Produced Conceptus Biochemistry

**DOI:** 10.1096/fj.202501962R

**Published:** 2025-08-19

**Authors:** Katheryn D. Peterson, Mary A. Oliver, Trevor F. Freeman, Shankar P. Poudel, Susanta K. Behura, David Kakhniashvili, Daniel L. Johnson, Rebecca R. Payton, Tulio M. Prado, Lew G. Strickland, J. Lannett Edwards, Elizabeth A. Shepherd, Jonathan E. Beever, Thomas E. Spencer, Daniel J. Mathew

**Affiliations:** ^1^ Department of Animal Science University of Tennessee Knoxville Tennessee USA; ^2^ School of Animal Sciences Virginia Polytechnic Institute and State University Blacksburg Virginia USA; ^3^ Division of Animal Sciences University of Missouri Columbia Missouri USA; ^4^ Proteomics and Metabolomics Core Facility University of Tennessee Health and Science Center Memphis Tennessee USA

**Keywords:** bovine, conceptus, endometrium, proteome, transcriptome

## Abstract

In vitro produced (IVP) bovine embryos enhance herd genetics for important food production traits and support advanced reproductive technologies. However, less than 50% survive the first month of gestation after transfer to the surrogate. While the biochemical consequences of in vitro culture on the embryo before transfer have been well documented, less is known after transfer when the embryo interacts with the endometrium and pregnancy failure occurs. To investigate crosstalk between the IVP and in vivo derived (IVD) bovine conceptus and endometrium, a Day 16 conceptus‐endometrial mono‐culture and co‐culture system was used, integrating transcriptomic and proteomic analyses. The IVP conceptus transcriptome (differentially expressed genes; DEG) diverged from the IVD conceptus but only after co‐culture with endometrium (377 DEG; FDR < 0.05). Further, of the IVP conceptus DEG, 81 genes (21%) were associated with abnormal embryonic or fetal development. Significant biological processes associated with abundant endometrial DEG induced by IVP conceptuses were related to GTPase activity and the inflammatory response (FDR < 0.01), the latter of which may be associated with increased MHC class II expression, which was specific to IVP conceptuses. Proteomic analysis of culture media identified 1031 and 604 differentially abundant proteins (FDR < 0.05) associated with the IVD and IVP conceptus‐endometrial co‐cultures, respectively, compared to endometrium alone, indicating the proteomic environment surrounding the IVP conceptus may be suboptimal. Collectively, compared to the IVD conceptus, evidence suggests the endometrium drives and responds to divergence of IVP conceptus biochemistry, likely contributing to pregnancy failure.

## Graphical abstract

IVD versus IVP bovine conceptus‐endometrial microenvironment. Compared to the in vivo derived (IVD) bovine conceptus, the in vitro produced (IVP) conceptus has a reduced number of transcribed genes, mRNA abundance for genes transcribed and increased expression of inflammatory mediators in response to the endometrium. In return, the endometrium increases expression of genes associated with the inflammatory response and decreases expression of genes involved in protein transport, limiting the protein microenvironment that supports conceptus growth and disrupting immune tolerance of pregnancy.

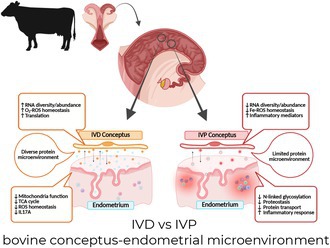

## 1 Introduction

In vitro produced (IVP) mammalian embryos have transformed reproductive technologies and research related to human fertility, wild animal conservation and agriculture. IVP bovine embryos are widely used to improve herd genetics for important food production traits, overcome infertility in repeat breeder animals, and support advanced reproductive technologies such as somatic cell nuclear transfer (SCNT) [1, 2, 3]. According to the international embryo technology society (IETS), global production of IVP bovine embryos has increased over 300% in the last 20 years [1], emphasizing the growing popularity of the technology and its potential to improve animal agriculture. In the clinic, in vitro produced embryos are routinely used to overcome human infertility and over 2.5 million in vitro fertilization (IVF) cycles are performed annually in women, resulting in 500 000 births per year [4].

Despite advantages, IVP embryos remain inferior in quality and developmental competence compared to their in vivo derived (IVD) counterparts. In vitro produced bovine embryos have an altered biochemistry in the form of DNA methylation, gene transcription, metabolism, lipid content, and cell division which contribute to reduced blastocyst formation before transfer to the recipient [5, 6, 7, 8, 9, 10]. Even under optimal culture conditions, IVP bovine embryo blastocyst formation rarely exceeds 40%, and only a fraction of IVP embryos are considered high quality [11, 12]. Post‐transfer survival rates are also poor, with fewer than 50% of fresh, high‐quality blastocysts surviving beyond Day 30 of gestation [12, 13, 14]. Based on a recent metanalysis, it was estimated that 59%–85% of transferred IVP bovine embryos fail to reach term [12]. Our understanding of IVP bovine embryo development after transfer to the recipient is limited, as the conceptus does not undergo advanced development, including conceptus elongation, in vitro.

Within the uterus, near Day 13 of development, the bovine conceptus begins to elongate in structure, enhancing crosstalk with the endometrium through secretion of conceptus secretory factors such as interferon tau (IFNT), the maternal recognition of pregnancy signal [15, 16]. By Day 16, the conceptus will extend approximately 60 mm in length yet is only 2 mm wide and remains unattached from the endometrium [15]. Conceptus elongation and the prolonged period of protracted development is supported by endometrial secretions collectively termed histotroph, produced largely by uterine glands within intercaruncular endometrium [15, 16]. Histotroph includes endometrial‐derived proteins, metabolites, ions, and amino acids as well as extracellular vesicles [15]. Conceptus attachment does not begin until near Day 20 gestation in cattle and is enhanced within the caruncular endometrium with formation of giant trophoblast binucleate cells that migrate through or fuse with the uterine epithelium [15]. Asynchronous or abnormal crosstalk between the conceptus and endometrium, particularly during elongation and conceptus attachment, is believed to contribute greatly to reproductive failure in cattle.

Recent studies suggest the endometrium can “sense” conceptus quality or competence as conceptus‐induced endometrial gene expression reflects methods of embryo production including IVF or SCNT [17, 18, 19, 20, 21]. Previously, we confirmed this using an elongated bovine conceptus‐endometrial co‐culture system coupled with endometrial RNA‐sequencing (RNA‐Seq) [17]. Major findings from the study suggested that IVP bovine conceptuses, within 6 h of co‐culture, induce an altered endometrial transcriptome reflective of a pro‐inflammatory state (increased expression of interleukin‐17A; *IL‐17A*) and reduced molecule transport via exocytosis compared IVD conceptuses, possibly impacting uterine histotroph and conceptus survival [17].

**FIGURE 1:**
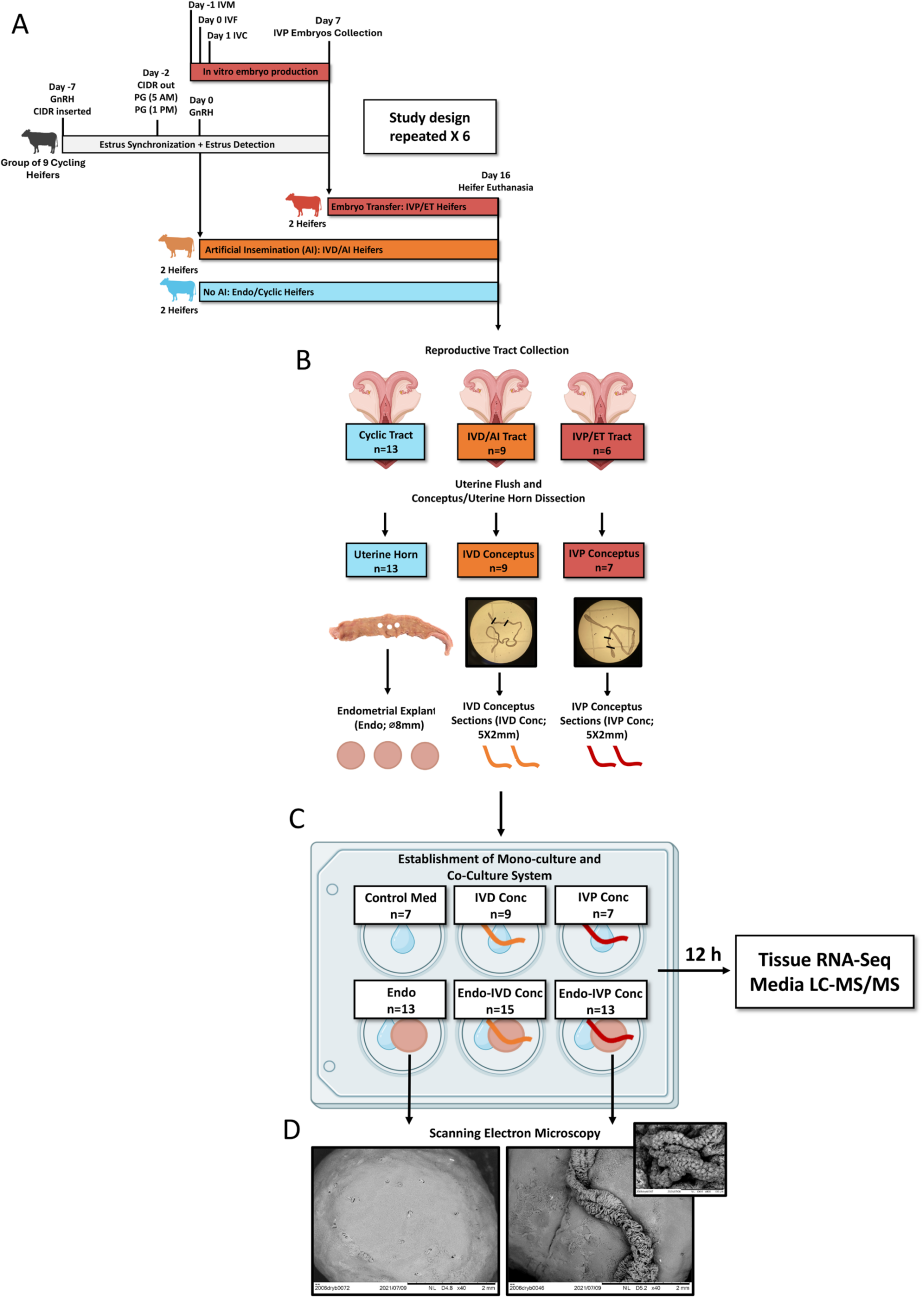
Experimental design. (A) To produce Day 16 IVD or IVP bovine conceptuses, Angus‐Holstein heifers underwent estrus synchronization and were bred by artificial insemination (AI) or received age and sire matched in vitro produced (IVP) embryos. A third group of synchronized heifers were not bred to produce cyclic Day 16 endometrium. (B) On Day 16 of the reproductive cycle or pregnancy, uteri were flushed with RPMI medium and intercaruncular endometrium and elongated conceptuses were used to produce multiple endometrial explants (⌀8 mm) and conceptus sections (5 × 2 mm), respectively. (C) In modified RPMI (2% dialyzed FBS and 1% antibiotic‐antimycotic), endometrial explant and conceptus sections were used to produce the following mono‐ and co‐cultures: (1) no tissue (Control Med; blank control; *n* = 7), (2) IVD conceptus section (IVD Conc; mono‐culture, *n* = 9), (3) IVP conceptus section (IVP Conc; mono‐culture, *n* = 7), (4) endometrial explant (Endo; mono‐culture, *n* = 13), (5) endometrial explant and IVD conceptus section (Endo‐IVD Conc; co‐culture, *n* = 15), and (6) endometrial explant and IVP conceptus section (Endo‐IVP Conc; co‐culture, *n* = 13). Cultures were incubated for 12 h in 5% CO_2_ and atmospheric O_2_ at 38.5°C before tissue and media were collected for subsequent RNA sequencing (RNA‐Seq) and liquid chromatography–tandem mass spectrometry (LC–MS/MS), respectively. (D) scanning electron microscopy (SEM) images of an Endo mono‐culture and Endo‐IVP Conc co‐culture.

In the present study, we took advantage of the elongating bovine conceptus structure and explant culture system to evaluate the impact of prolonged IVD and IVP conceptus crosstalk on endometrium as well as conceptus transcriptomes and associated media proteomes (Figure 1). To establish the culture system, Day 16 elongated IVD and IVP conceptuses were sectioned and Day 16 cyclic heifer intercaruncular endometrium biopsied to establish extended (12 h) tissue monocultures and co‐cultures. During each tissue culture repetition, the system allowed us to evaluate the impact of both IVD and IVP conceptuses on endometrium from a single uterus and likewise, the impact of monoculture and co‐culture on an individual conceptus transcriptome and associated proteome. Given our previous findings, it was hypothesized that extended crosstalk between the IVP bovine conceptus and endometrium would result in suboptimal histotroph within the co‐culture microenvironment in the form of protein and aberrant endometrial immunology compared to the IVD conceptus.

## 2 Materials and Methods

### 2.1 Description of Experimental Design

This study is a continuation of a larger study investigating crosstalk between the Day 16 elongating bovine conceptus and endometrium [22]. A detailed description of animal work, tissue culture, tissue transcriptomics (RNA‐Seq) and media proteomics (LC–MS/MS) is provided by Peterson et al. (2025) [22]. Study procedures involving animals were conducted in accordance with the University of Tennessee, Knoxville, Institutional Animal Care and Use Committee (IACUC). To produce Day 16 cyclic endometrium or Day 16 IVD and IVP conceptuses, groups of Angus‐Holstein heifers underwent estrus synchronization and either remained cyclic (not‐bred), were bred by artificial insemination (AI; IVD conceptuses) or received IVP bovine embryos (Day 7‐embryo transfer) age and sire matched with IVD conceptuses (Figure 1). On Day 16 of the estrous cycle or pregnancy, heifer uteri were collected and flushed with Roswell Park memorial institute (RPMI) medium. After, cyclic uteri were used to produce multiple endometrial explants and flushed IVD and IVP conceptuses, multiple conceptus sections. Explants and conceptus sections were subsequently cultured independently (mono‐culture) or in co‐culture (IVD or IVP conceptus section overlaying endometrium) for 12 h in a modified RPMI containing heat inactivated 2% dialyzed fetal bovine serum and 1% anti‐biotic, anti‐mycotic. After, endometrial and conceptus tissue were collected for RNA‐Seq and media for LC–MS/MS proteomic analysis to identify transcripts and proteins dependent on tissue crosstalk and conceptus origin (IVP compared to IVD). In total, 13 cyclic uteri were used to produce the endometrial explants and 9 IVD and 7 IVP conceptuses were utilized to produce conceptus tissue sections. In this way, endometrium from the same heifer and conceptus tissue from the same conceptus (IVD or IVP) could be utilized as a crosstalk control and included in mono‐culture and co‐culture treatments. A complete list of endometrial explants, conceptus sections and the heifer from which the tissue was derived are provided in Data S1.

### 2.2 In Vitro Embryo Production

The IVP bovine embryos were derived as previously described [23]. Briefly, bovine cumulus‐oocyte complexes (COCs) were isolated from ovaries obtained from a local abattoir (Southeastern Provision, Bean Station, TN). Using an ovarian slicing and washing method, bovine oocytes were collected into oocyte collection medium and oocytes with multiple layers of compact cumulus cells and dark, homogenous ooplasm were placed into 500 μL of oocyte maturation medium in groups of 25–40. The COC were matured at 38.5°C in 5.5% CO_2_ and humidified air for 24 h. To achieve IVF, frozen–thawed sperm from an Angus bull with proven fertility were washed and added (7.5 × 10^5^) to the oocytes for 18 h. Sperm from this bull was also used to produce sire matched elongated IVD conceptuses in heifers that underwent AI. The next day, the surrounding cumulus cells were removed from the presumptive zygotes (PZ) by vortex and the PZ were cultured in groups of 20–40 in 500 μL of potassium simplex optimized medium (KSOM) at 38.5°C in 5.5% CO_2_ and 7% O_2_ [24, 25]. Embryo cleavage was assessed at 72 h and on Day 7 of development, two fresh grade 1 blastocysts underwent ET into estrus‐synchronized recipient heifers to produce elongated IVP conceptuses.

### 2.3 Estrous Synchronization, Endometrial Explants and Conceptus Sections

Estrous cycles of groups of pubertal cross‐bred heifers were synchronized using a 5‐Day Co‐Synch estrous synchronization protocol involving vaginal progesterone (P4) supplementation (controlled internal drug release; CIDR). As recommended for beef heifers, the protocol included two doses of prostaglandin (PG), one at time of CIDR removal and a second, 8 h later (Figure 1). Fifty‐eight hours after the final gonadotropin releasing hormone (GnRH) injection and CIDR removal, estrus was confirmed (ESTROTECT) and heifers were either bred by AI using frozen–thawed semen from an Angus bull with proven fertility (same bull used during IVF) or were not bred by AI. Bred (IVD/AI) and non‐bred (Endo/Cyclic) heifers were utilized to produce elongated IVD bovine conceptuses and synchronized cyclic endometrium, respectively. As indicated above, to produce age and sire matched IVP conceptuses, a third group of synchronized non‐bred heifers (IVP/ET) underwent ET on Day 7 of the estrous cycle with two IVP bovine blastocysts to account for early embryonic mortality. On Day 16, heifers were humanely euthanized by captive bolt at a local processing facility (Southeastern Provisions) and the reproductive tracts were placed on ice for transport to the laboratory (45 min). Considering ovulation in beef heifers occurs approximately 66 h after CIDR removal and it takes approximately 10 h for sperm to reach the oviduct and undergo capacitation, we considered fertilization to occur approximately 68 to 72 h after CIDR removal (10–14 h after AI) [26, 27]. Thus, IVF in production of IVP embryos was timed accordingly in the laboratory so that conceptuses were of similar developmental age and morphological stage. This was confirmed based on morphological appearance and extensive elongation of IVD and IVP conceptus on Day 16, when heifer uteri were collected (Figure 1). Considering the number of corpora lutea (CL) in AI heifers, transferred embryos in ET heifers and number of elongated IVD and IVP conceptuses collected, the Day 16 pregnancy rate for AI heifers was 70% whereas, 47% of IVP conceptuses survived to Day 16. In total, endometrial explants were produced from 13 cyclic heifers and 9 and 6 heifers produced 9 and 7 IVD and IVP conceptuses, respectively, during the study.

### 2.4 Establishment of Conceptus and Endometrial Tissue Cultures

Methods related to tissue cultures are described in detail by Peterson et al. (2025) [22]. Cyclic and pregnant reproductive tracts were rinsed with Dulbecco's phosphate buffered saline (DPBS; Gibco) and ovaries were evaluated for the presence of a mid‐luteal phase CL [28]. Both uterine horns were flushed with 20 mL of RPMI medium (Gibco) to collect Day 16 cyclic and pregnant uterine flush fluid (UFF) and elongated IVD and IVP conceptuses. The UFF from the uterine horn ipsilateral to the CL also underwent proteomic analysis. To establish the tissue culture system, intercaruncular endometrial explants were dissected from the middle third of the ipsilateral uterine horn using sterile biopsy punches (⌀8 mm) and conceptuses were dissected into 5 × 2 mm sections using sterile scalpel blades. The tissues were then added to 1 mL of RPMI medium supplemented with heat inactivated 2% dialyzed fetal bovine serum (FBS; Gibco) and 1% antibiotic‐antimycotic (ABAM; Gibco) in 24 well plates (CELLSTAR; Greiner). The following cultures in media were prepared: (1) no tissue (Control Med; *n* = 7), (2) IVD conceptus section (IVD Conc; mono‐culture, *n* = 9), (3) IVP conceptus section (IVP Conc; mono‐culture, *n* = 7), (4) endometrial explant (Endo; mono‐culture, *n* = 13), (5) endometrial explant and IVD conceptus section (Endo‐IVD Conc; co‐culture, *n* = 15), and (6) endometrial explant and IVP conceptus section (Endo‐IVP Conc; co‐culture, *n* = 13). Cultures were incubated for 12 h in 5% CO_2_ and atmospheric O_2_ at 38.5°C. After, endometrial and conceptus tissue as well as culture media were collected for subsequent transcriptomic and proteomic analysis, respectively. Scanning electron microscopy (SEM) images of an endometrial explant and an Endo‐IVP Conc culture were taken with a Hitachi Tabletop Microscope (TE3030). The tissues were imaged fresh and without fixation.

### 2.5 Tissue RNA Extraction and RNA‐Sequencing

Endometrial explant and conceptus RNA extraction was conducted as previously described in detail [22]. Briefly, endometrial and conceptus total RNA was extracted using bead homogenization (Beadmill 4; FisherScientific) and Trizol following kit on‐column isolation (endometrium) or simply the latter (conceptus) with a DNase I digestion step (RNeasy; Qiagen). Eluted RNA was assessed for quantity and quality using a NanoDrop 1000 (ThermoFisher) and the 2100 Bioanalyzer (Agilent Technologies), respectively. Total RNA was extracted from endometrial tissue from the following cultures: Endo (*n* = 13), Endo‐IVD Conc (Endo‐IVD Conc; *n* = 15), and Endo‐IVP Conc (Endo‐IVP Conc; *n* = 12) and total RNA was extracted from conceptus tissue from the following cultures: IVD Conc (*n* = 7), IVP Conc (*n* = 7), Endo‐IVD Conc (Endo‐IVD Conc; *n* = 9), and Endo‐IVP Conc (Endo‐IVP Conc; *n* = 7). Endometrial and conceptus tissue RNA integrity numbers (RIN) ranged from 5.2 to 9.3 and 6.5 to 10, respectively. Tissue total RNA underwent RNA‐Seq as described in detail by Peterson et al. (2025) [22]. Briefly, library preparation including ploy (A) enrichment, amplification and sequencing [NovaSeq 6000 (Illumina)] was performed at the University of Missouri DNA Core Facility (Columbia, MO). The RNA‐Seq libraries were prepared using the Stranded RNA Prep Ligation Kit (Illumina) and final amplified libraries were purified by addition of Axyprep Mag PCR Clean‐up beads (Corning). The RNA‐Seq raw sequences (fastq) underwent adaptor removal and quality trimming using Trimmomatric and quality pair‐end reads were aligned to the bovine reference genome (ARS‐UCD1.2.103) using the STAR and Hisat2 programs for the endometrium and conceptus tissues, respectively [29]. The sorted binary alignment maps and the NCBI annotation assembly were subject to featureCounts to quantify read counts of genes for each sample. The number of fragments per kilobase of transcript per million mapped reads (FPKM) were calculated from the read count data and gene length using edgeR. Raw fastq and featureCount data are available within the national center for biotechnology information (NCBI) Gene Expression Omnibus (GEO; accession numbers: GSE256172 and GSE295732).

### 2.6 Real‐Time Quantitative PCR (RT‐qPCR)

Explant RNA was assayed by RT‐qPCR to verify endometrial response to the conceptus prior to RNA‐Seq. During the RT‐qPCR, previously validated conceptus‐induced, IFNT dependent and independent DEG were assayed as previously described in detail [16, 17, 30]. Briefly, 500 ng of total RNA was reverse transcribed into cDNA using the High‐Capacity cDNA Reverse Transcription Kit (Applied Biosystems; ThermoFisher). Seven serial dilutions (1:4) were made from a resulting sample cDNA pool and used to determine percent primer efficiencies (*E* = [10^(−1/slope)^−1]/2*100). A dissociation analysis was included for each primer set and gel electrophoresis was used to validate amplicon length. Individual cDNA samples were diluted 1:20 in nuclease free water and 20 μL sample RT‐qPCR reactions were prepared in duplicate with 10 μL SYBR Green (Applied Biosystems), 1.2 μL of the forward primer, 1.2 μL of the reverse primer, 2.6 μL of nuclease‐free water, and 5 μL of cDNA template (6.25 ng mRNA equivalent). During RT‐qPCR and for each target gene, sample cDNA, no‐reverse transcriptase (NRT), and no‐template controls (NTC; cDNA replaced with nuclease free water) were assayed on a single 96‐well plate. Thermocycler (QuantStudio 3; Applied Biosystems) settings were 50°C for 2 min, 95°C for 2 min, followed by 40 cycles at 95°C for 15 s and 60°C for 1 min. Eight reference genes were assayed across a subset of cDNA samples to identify reference genes [31]. Tyrosine 3‐monoxygenase (*YWHAZ*) and ring finger protein 11 (*RNF11*) were the most stably expressed genes across all samples and were used to normalize target gene relative expression using a generalized delta–delta cycle method (∆∆Cq, known also as ∆∆Ct) [32]. Prior to statistical analysis, all normalized relative gene expression quantities were log transformed (Log10). A list of target genes, primer sequences, primer efficiencies and corresponding amplicon sizes associated with the RT‐qPCR analysis can be found in Table S1.

### 2.7 Culture Medium Proteomic Analysis

Media samples collected from the cultures were analyzed for proteomic composition using LC–MS/MS at the University of Tennessee Health Sciences Center, Proteomics and Metabolomics Core (UTHSC PMC; Memphis, TN) as previously described in detail [22]. Media from the following cultures was assayed: (1) Control Med (*n* = 7), (2) IVD Conc (*n* = 7), (3) IVP Conc (*n* = 7), (4) Endo (*n* = 9), (5) Endo‐IVD Conc (*n* = 10) and (6) Endo‐IVP Conc (*n* = 10). The UFF from Endo/Cyclic heifers (*n* = 4) and heifers carrying IVD (IVD/AI; *n* = 4) and IVP (IVP/ET; *n* = 5) conceptuses were also assayed. In preparation of proteomics, samples were normalized to 25 μg of protein in 50 μL (culture media) or 160 μL (UFF) using RPMI medium and supplemented with SDS and triethylammonium bicarbonate (TEAB) to the final concentrations of 0.5% and 100 mM (pH 8.3), respectively. Sample proteins were precipitated with 5 volumes of cold acetone and protein pellets were washed, air dried and redissolved (50 μL of digestion buffer; 100 mM ammonium bicarbonate). Dissolved proteins were digested (1 μg of Pierce trypsin/Lys‐C protease mixture; ThermoFisher) and peptide samples were desalted using Pierce C‐18 spin tips (ThermoFisher) according to the manufacturer's protocol, and vacuum dried.

Each peptide sample (10 μg) was re‐dissolved in 100 μL of loading buffer and 5 μL (0.5 μg) was analyzed using LC–MS/MS method with 160 min LC gradient for peptide/protein identification and label‐free quantification (LFQ). Raw MS data were acquired on an Orbitrap Fusion Lumos mass spectrometer (ThermoFisher) operating in line with Ultimate 3000RSLCnano UHPLS system (ThermoFisher). The peptides were trapped on an Acclaim PepMap 100 nanoViper column (75 μm × 20 mm; ThermoFisher), washed with loading buffer and separated on an Acclaim PepMap RSLC nanoViper column (75 μm × 500 mm, C‐18, 2 μm, 100 Å; ThermoFisher) using water and acetonitrile with 0.1% formic acid as solvents A and B, respectively. The following multi‐point linear gradient was applied: 3% B at 0–4 min, 5% B at 5 min, 23% B at 110 min, 30% B at 120 min, 90% B at 123–133 min, and 3% B at 136–160 min. Data dependent acquisition (DDA) method was used where full MS scans were performed in the Orbitrap analyzer at 120 000 (FWHM, at m/z = 200) resolving power to determine the accurate masses (m/z) and the abundances of peptides. The data dependent MS2 analysis was performed on precursor ions with peptide‐specific isotopic pattern, charge state 2–6, and intensity of at least 10 000 to determine peptide sequences. For MS2 scans, peptide ions were isolated using quadrupole isolation with 0.7 m/z window, fragmented (HCD, 30% NCE), and the fragment masses were determined in the Orbitrap analyzer at 30 000 (FWHM, at m/z = 200) resolving power. Dynamic exclusion was applied for 45 s.

Analysis of the raw MS data was performed with Proteome Discoverer 2.4 (ThermoFisher) using Sequest HT search algorithm and bovine protein databases (SwissProt, *Bos taurus* , Tax‐ID 9913, v.04‐30‐2022, 6016 entries; TrEMBL, *Bos taurus* , Tax‐ID 9913, v.04‐30‐2022, 40 736 entries). The precursor and fragment ion mass tolerances were set to 10 ppm and 0.02 Da, respectively, and raw data were filtered for the precursor ions with S/N of at least 1.5. The peptide spectrum matches (PSM) were filtered for further analysis using a delta Cn (normalized score distance from the second‐best‐scoring PSM) threshold of 0.05 and q‐values were calculated at PSM level (Percolator), and then, at peptide level (Qvality algorithm) to control false discovery rate (FDR). The FDR threshold of 0.01 was used to validate and filter the data at PSM and then at peptide levels. Identified candidate protein sum‐PEP scores were used to calculate the experimental *q*‐values at protein level. The candidate proteins were further validated (without filtering) using 0.01 and 0.05 FDR thresholds. Peptide quantification was based on LC peak area with at least 5 data‐points. The Proteome Discoverer protein abundances were determined as summed abundances of assigned peptides and default imputation was used for missing values (assigned zero). Unique and razor peptides were used for protein quantification.

### 2.8 Statistical Analyses

The RNA‐seq and LC–MS/MS data were statically analyzed as described by Peterson et al. (2025) [22]. For the RNA‐Seq data, to potentially identify unique gene transcripts within the tissues in general and without direct comparisons, transcripts detected in 70% of the endometrial and conceptus samples within a treatment and with an average FPKM ≥ 0.05 are presented in Data S2 and S3, respectively. In preparation of direct comparisons, featureCount output data were independently filtered at an alpha level of 0.01 with the DESeq2 package and unexpected variation, unrelated to treatment, was accounted for using the RUVSeq package [33, 34]. Differentially expressed genes (DEG) between tissues were determined after multiple comparison correction (Benjamini‐Hochberg) and P‐value adjustment (FDR; *α* ≤ 0.05) using the DESeq function. Quantified peptide LC–MS/MS data were normalized with the Cyclic Loess normalization function in the R/Bioconductor package limma and analyzed to determine differential expression [35]. An ad hoc *t*‐test and fold change were calculated using an ANOVA for all pairwise comparisons between treatments. A Tukey correction was applied and only proteins with *p* < 0.05 were considered differentially abundant proteins (DAP). To better identify tissue proteins with a likelihood of secretion, identified proteins (DAP) found to be more abundant in cultures underwent GO cellular component complete annotation using the Gene Ontology Resource (Release: 2024‐11‐03) powered by Panther (19.0). The *Bos taurus* background was used and proteins predicted to reside within the extracellular region (GO:0005576) were considered to have a high likelihood of secretion. Using the list of genes identified as expressed within the endometrium and conceptus tissue based on FPKM values, the same method was used to identify potential endometrial and conceptus cell plasma membrane proteins (GO:0005886) which may serve as ligands or receptors for secreted proteins. All protein data associated with tissue cultures, possible secreted proteins and tissue ligands/receptors, and identified UFF proteins are presented in Data [Link], [Link], [Link], respectively.

Principal component analysis (PCA) plots and heatmaps (DEG and/or Pearson Correlation) associated with the transcriptomic and proteomic data were generated using the prcomp function in R on the 1000 most variable transcripts or proteins and the ggplot2 and ComplexHeatmap visualization packages [36]. Venn diagrams were plotted to compare DEG and DAP between treatment conditions using Venny 2.1 (BioinfoG) as well as UFF proteins, the latter of which is presented in Figure S1. The Data for Annotation, Visualization, and Integrated Discovery (DAVID) Bioinformatics Resource was utilized to perform gene ontology (GO) analyses of DEG and DAP using the *Bos Taurus* background [37]. Enriched biological processes (BP; GO direct category) and Kyoto Encyclopedia of Genes and Genomes (KEGG) pathways were analyzed [37]. The GO direct BP and KEGG pathways were considered significant when FDR ≤ 0.05 or tendency for significance when 0.05 < FDR ≤ 0.10. The mouse genome informatics (MGI; version 6.24) database Analysis Tool (batch query) was used to identify IVP conceptus DEG associated with an abnormal embryonic or fetal mammalian phenotype. The RT‐qPCR log transformed data were statistically analyzed using a generalized linear model (Proc GLM; SAS 9.4). Normality of residual data were assessed using the diagnostics residuals statement and histogram. Data are presented as the least squares means (LSM) ± standard error of the LSM (SEM) (Table S2).

## 3 Results

### 3.1 The Endometrial Transcriptome in Response to IVP and IVD Conceptuses

**FIGURE 2:**
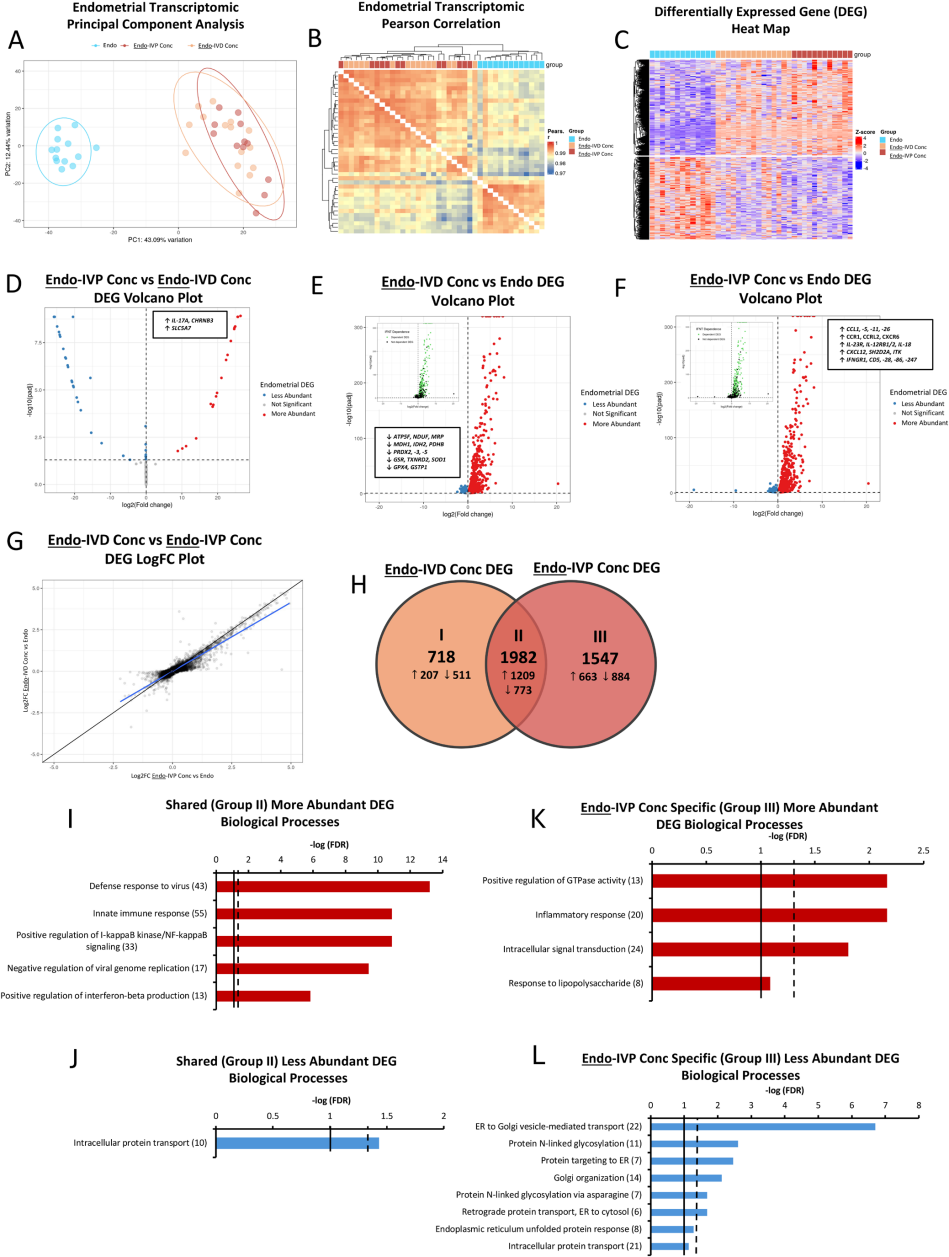
Endometrial transcripts identified by RNA sequencing (RNA‐Seq). (A–C) Principal component analysis (PCA; A), Pearson correlation heat map (B) and differential expressed gene (DEG) heat map (C) of endometrial transcripts from mono‐ and co‐culture endometrium identified by RNA‐Seq [Endo (*n* = 13), Endo‐IVD Conc (*n* = 15), or Endo‐IVP Conc (*n* = 12)]. (D) Endo‐IVP Conc compared to Endo‐IVD Conc DEG volcano plot. (E, F) Endo‐IVD Conc (E) or Endo‐IVP Conc DEG (F) volcano plot compared to Endo. Small internal graph left of center in (E and F) are the same graph with IFNT dependent DEG highlighted green. (G) Endo‐IVD Conc (y‐axis) and Endo‐IVP Conc DEG (x‐axis) log fold change (LogFC) plot associated with DEG volcano plots (E and F), respectively. (H) Venn diagram of Endo‐IVD Conc and Endo‐IVP Conc DEG identified when compared to mono‐culture Endo. Arrows indicate the status of DEG expression (more or less abundantly expressed). (I, J) Biological processes (BP) associated with more (1209; I) or less (773; J) abundantly expressed DEG shared between Endo‐IVD Conc and Endo‐IVP Conc (Venn diagram H, Group II). (K, L) Biological processes associated with more (663) or less (884) abundantly expressed DEG specific to Endo‐IVP Conc (Venn diagram H, Group III). Broken and solid line within (I‐J) indicate FDR = 0.05 and 0.10, respectively. The number at the end of the BP indicates the number of DEG involved in the pathway.

RNA‐seq was used to identify mono‐culture and co‐culture conceptus and endometrial gene transcripts. Based on the principal component analysis (PCA) and Pearson correlation and differentially expressed gene (DEG) heatmaps, gene transcripts in endometrium co‐cultured with IVD and IVP conceptus (Endo‐IVD Conc and Endo‐IVP Conc, respectively) tissue were distinct from that of mono‐cultured endometrium (Endo; Figure 2A–C). Endo‐IVP Conc endometrium contained 22 more and 33 less abundantly expressed DEG compared to the Endo‐IVD Conc (Figure 2D). Transcripts for *IL‐17A*, cholinergic receptor nicotinic beta 3 (*CHRNB3*) and a choline transporter, solute carrier family 5 member 7 (*SLC5A7*), were between 22 and 25‐fold greater in Endo‐IVP Conc endometrium (Data S2). Compared to the Endo, 2700 (*n* = 1416 more‐ and 1284 less abundantly expressed) and 3529 (*n* = 1873 more‐ and 1656 less abundant expressed) DEG were identified in the Endo‐IVD Conc and Endo‐IVP Conc endometrium, respectively (FDR ≤ 0.05; Figure 2C).

To determine if maternal recognition (IFNT signaling) was impaired between the two conceptus types, possibly contributing to the DEG discrepancy, a previously published list of bovine IFNT regulated genes (IRG) was used to identify IFNT dependent DEG in the Endo‐IVD Conc and Endo‐IVP Conc endometrium [17]. Regardless of conceptus type, endometrial expression of IRG was similar with the same 375 more abundantly expressed IRG and a similar number of less abundantly expressed IRG (20 and 15, Endo‐IVD and Endo‐IVP Conc, respectively) with comparable average log fold changes (LogFC). Of note, out of the top 30 most abundantly expressed DEG in Endo‐IVD and Endo‐IVP Conc endometrium compared to Endo, 29 and 27 DEG, respectively, were IRG and had an average LogFC > 6 (Figure 2E,F). Thus, the discrepancy in the number of DEG (*n* = 2700 vs. 3529) between Endo‐IVD Conc and Endo‐IVP Conc endometrium compared to Endo does not appear to be related to IFNT, the maternal recognition of pregnancy signal. Mean abundances of IFNT protein (IFNT3) were also similar in IVD and IVP Conc mono‐culture and co‐culture media (Data S4).

Using the complete list of DEG, a Venn diagram was plotted to compare Endo‐IVD and Endo‐IVP Conc endometrial DEG and identify biological processes (BP) or Kyoto Encyclopedia of Genes and Genomes (KEGG) pathways specific to conceptus type (Figure 2H–L). Fourteen BP and 25 KEGG pathways (FDR ≤ 0.05), mostly related with IFN signaling, were associated with the shared more abundantly expressed (*n* = 1209) endometrial DEG (Figure 2I). The BP intracellular protein transport (Figure 2J) and 10 KEGG pathways including metabolic pathways, glycosaminoglycan biosynthesis and oxidative phosphorylation (FDR ≤ 0.05) were associated with the shared less abundantly expressed (*n* = 773) endometrial DEG. No BP or KEGG pathways were associated with the more abundantly expressed DEG specific to Endo‐IVD Conc endometrium (*n* = 207), however, 17 KEGG pathways (FDR ≤ 0.05) were associated with the less abundantly expressed DEG (*n* = 511) including chemical carcinogenesis related to reactive oxygen species (ROS) (FDR < 0.001) and oxidative phosphorylation (FDR < 0.001), which were the third and fifth most significant, respectively.

Three BP were associated with the more abundantly expressed DEG specific to Endo‐IVP Conc endometrium (*n* = 663) including, from most to least significant, positive regulation of GTPase activity (FDR < 0.01), inflammatory response (FDR < 0.01), and intracellular signal transduction (FDR < 0.05). Response to lipopolysaccharide (LPS) tended to be significant (FDR = 0.08; Figure 2K). Genes associated with inflammatory responses and response to LPS included several chemokines, cytokines, and related receptors associated with increased T‐helper type 1 lymphocyte (Th1), Th17 and natural killer (NK) cell activity including CC motif chemokine ligands (*CCL1*, *CCL5*, *CCL11* and *CCL26*), CC and CXC ligand receptors (*CCR1*, *CCRL2*, and *CXCR6*), interleukin‐23 receptor (*IL‐23R*), IL‐12 receptor B1 and B2 (*IL‐12RB*), and *IL‐18*. Seven BP and five KEGG pathways (FDR ≤ 0.05) were associated with less abundantly expressed DEG specific to Endo‐IVP Conc endometrium (*n* = 884), several of which were associated with some aspect of proteostasis and protein transport including the most significant BP (ER to Golgi vesicle‐mediated transport; *p* < 0.001) and KEGG pathway (Protein processing in endoplasmic reticulum; *p* < 0.001) (Figure 2L). The second most significant down‐regulated BP and KEGG pathway was related to protein glycosylation (*p* < 0.01). Endometrial DEG associated with each tissue and comparison are provided in Data S2.

### 3.2 IVD and IVP Conceptus Transcriptomes in Response to Endometrium

**FIGURE 3:**
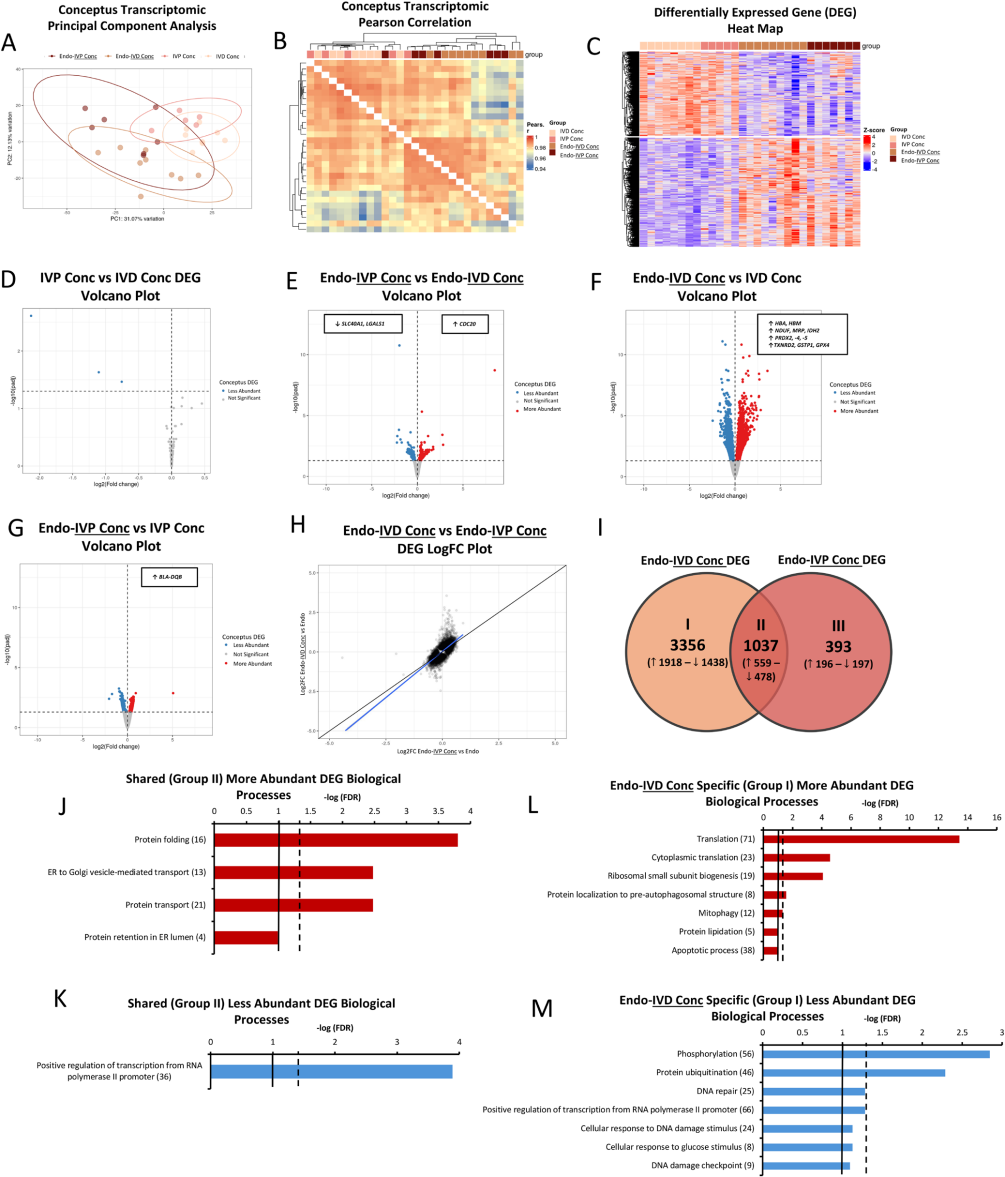
Conceptus transcripts identified by RNA sequencing (RNA‐Seq). (A–C) Principal component analysis (PCA; A), Pearson correlation heat map (B) and differential expressed gene (DEG) heat map (C) of conceptus transcripts from mono‐ and co‐culture conceptus tissue identified by RNA‐Seq [IVD Conc (*n* = 7), IVP Conc (*n* = 7), Endo‐IVD Conc (*n* = 9), or Endo‐IVP Conc (*n* = 7)]. (D) IVP Conc compared to IVD Conc DEG volcano plot. (E) Endo‐IVP Conc compared to Endo‐IVP Conc DEG volcano plot. (F, G) Endo‐IVD Conc compared to IVD Conc (F) and Endo‐IVP Conc compared to IVP Conc (G) DEG volcano plots. (H) Endo‐IVD Conc (y‐axis) and Endo‐IVP Conc DEG (x‐axis) log fold change (LogFC) plot associated with DEG volcano plots F and G, respectively. (I) Venn diagram of Endo‐IVD Conc and Endo‐IVP Conc DEG identified when compared to mono‐culture IVD Conc and IVP Conc tissue, respectively. Arrows indicate the status of DEG expression (more or less abundantly expressed). (J, K) Biological processes (BP) associated with more (559; J) and less (478; K) abundantly expressed DEG shared between Endo‐IVD Conc and Endo‐IVP Conc (Venn diagram I, Group II). (L, M) Biological processes associated with more (1918; L) and less (1438; M) abundantly expressed DEG specific to Endo‐IVD Conc (Venn diagram I, Group I). Broken and solid line within (J–M) indicate FDR = 0.05 and 0.10, respectively. The number at the end of the BP indicates the number of DEG involved in the pathway.

The PCA and Pearson correlation and DEG heatmaps suggested mono‐cultured IVD and IVP conceptus (IVD and IVP Conc, respectively) transcriptomes were similar, though diverged during co‐culture with endometrium (Figure 3A–C). Compared to mono‐cultured IVD Conc tissue, only three DEG (all less abundantly expressed) were identified in mono‐cultured IVP conceptus tissue (IVP Conc; Figure 3D). However, when the co‐cultured tissues were compared, 377 (*n* = 215 more‐ and 162 less‐abundantly expressed) DEG were identified in IVP conceptus tissue (Endo‐IVP Conc; Figure 3E). Three KEGG pathways (FDR ≤ 0.05) related to various diseases (Epstein–Barr virus infection, Parkinson's and viral carcinogenesis) were associated with more abundantly expressed DEG in Endo‐IVP Conc tissue. One KEGG pathway (metabolic pathways; *p* < 0.05) was associated with less abundantly expressed DEG. When DEG underwent mammalian phenotype analysis using the mouse genome informatics (MGI) database, 56 more‐ (*n* = 215/56; 26%) and 25 less‐abundantly expressed (*n* = 162/25; 15%) DEG in Endo‐IVP Conc tissue were associated with abnormal embryonic and/or fetal development including embryonic lethality. Further, when Endo‐IVD Conc tissue (co‐culture) were compared to IVD Conc tissue (mono‐culture) the number of DEG (> 3‐fold) and LogFC difference in gene expression was considerably greater than that IVP conceptus tissue (Figure 3F–H).

When comparing Endo‐IVD Conc and Endo‐IVP Conc DEG induced by co‐culture (Figure 3I), a total of 3356 (*n* = 1918 more‐ and 1438 less‐abundantly expressed) and 393 (*n* = 196 more‐ and 197 less‐abundantly expressed) DEG were identified as specific to Endo‐IVD Conc and Endo‐IVP Conc tissue (> 8.5 fold difference in number of unique transcripts), respectively (Figure 3I). Three BP and 13 KEGG pathways, mostly related to protein processing and transport, were found to be associated with more abundantly expressed (*n* = 559) DEG shared between Endo‐IVD Conc and Endo‐IVP Conc tissue (Figure 3J). The most significant BP was protein folding (*p* < 0.001) whereas the most significant KEGG pathway was protein processing in the ER (*p* < 0.001). KEGG pathways hypoxia inducible factor‐1 signaling (*p* < 0.01) and metabolic pathways (*p* < 0.05) were the second and third most significant. Positive regulation of transcription from RNA polymerase II promotor was the only BP associated with the shared less abundantly expressed (*n* = 478) conceptus DEG (*p* < 0.001; Figure 3K).

Five BP and 19 KEGG pathways were associated with the more abundantly expressed DEG specific to Endo‐IVD Conc tissue (*n* = 1918) including the most significant BP and KEGG pathway, translation (FDR < 0.001) and the ribosome (FDR < 0.001), respectively (Figure 3L). Likewise, four BP and four KEGG pathways were associated with the less abundantly expressed DEG (*n* = 1438) including the most significant BP and KEGG pathway, phosphorylation (FDR < 0.01) and the cell cycle (FDR < 0.001), respectively (Figure 3M). Of note, hemoglobin subunit alpha 1 (*HBA*) was the most abundantly expressed DEG specific to Endo‐IVD Conc tissue. No BP or KEGG pathways were associated with the more or less abundantly expressed DEG specific to Endo‐IVP Conc tissue, however, several genes related to cancer progression and immune recognition including a major histocompatibility (MHC) Class II molecule, bovine leukocyte antigen (BLA)‐DQB, were identified as a top 15 most abundantly expressed DEG specific to IVP conceptus tissue co‐cultured with endometrium. Conceptus DEG associated with each tissue and comparison are provided in Data S3.

### 3.3 Mono‐Culture IVD and IVP Conceptus Media Protein

**FIGURE 4:**
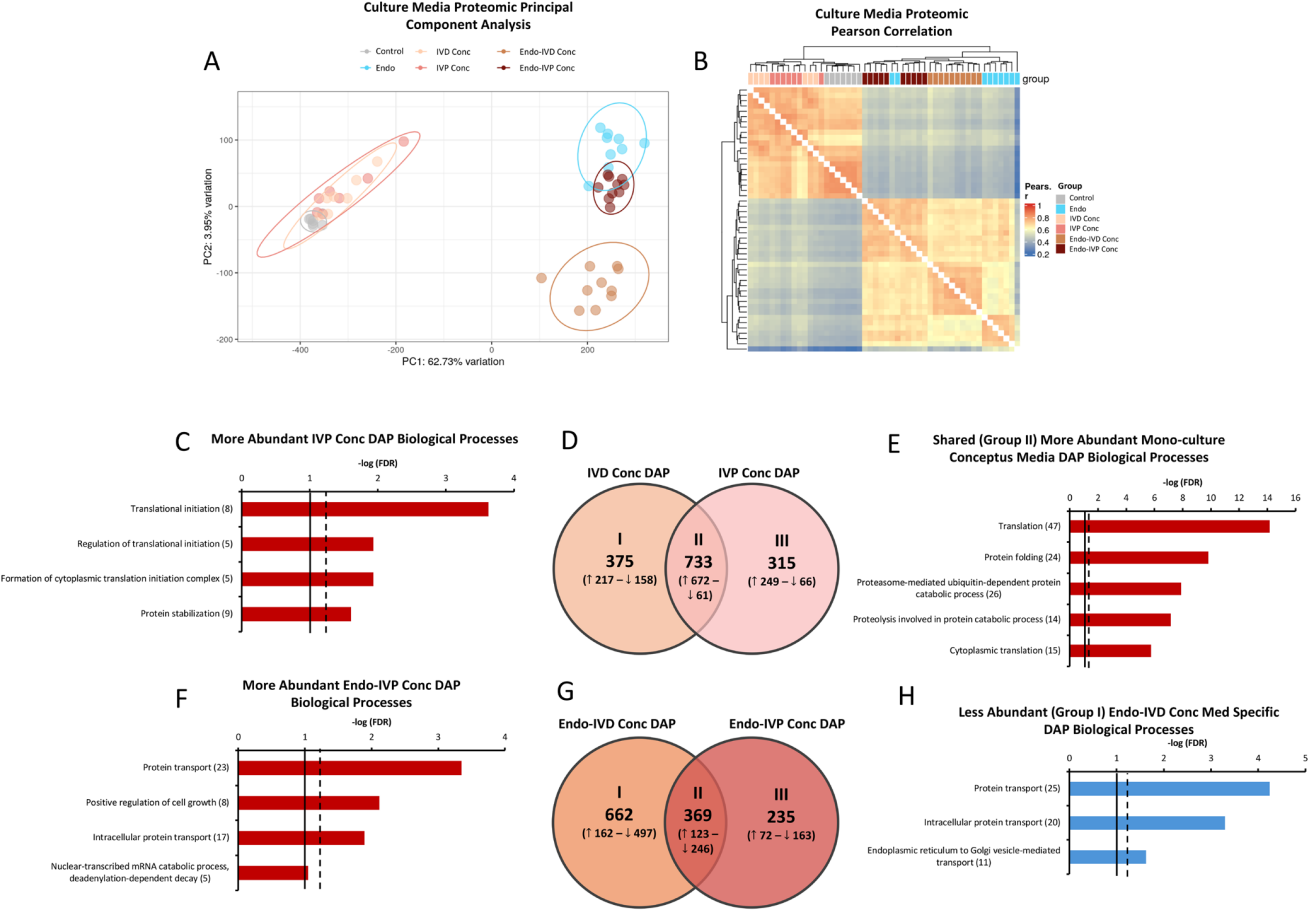
Mono‐ and co‐culture media protein identified by liquid chromatography–tandem mass spectrometry (LC–MS/MS). (A, B) Principal component analysis (PCA; A) and Pearson correlation heat map (B) of blank control and mono‐ and co‐culture media protein identified by LC–MS/MS [Control Med (blank control; *n* = 7), IVD Conc (*n* = 7), IVP Conc (*n* = 7), Endo (*n* = 9), Endo‐IVD Conc (*n* = 10), or Endo‐IVP Conc (*n* = 10)]. (C) Biological processes associated with more abundant IVP Conc mono‐culture differentially abundant protein (DAP) compared to IVD Conc mono‐cultures. (D) Venn diagram of mono‐culture IVD Conc and IVP Conc DAP identified when compared to Control Med. (E) Biological processes associated with more abundant (672; Venn diagram D; Group II) DAP shared between IVD Conc and IVP Conc mono‐cultures. (F) Biological processes associated with more abundant Endo‐IVP Conc co‐culture DAP compared to Endo‐IVD Conc co‐cultures. (G) Venn diagram of co‐culture Endo‐IVD Conc and Endo‐IVP Conc DAP identified when compared to Endo mono‐cultures. (H) Biological processes associated with less abundant (497; Venn diagram G; Group I) DAP specific to Endo‐IVD Conc co‐cultures. Broken and solid line within C, E, F and H indicate FDR = 0.05 and 0.10, respectively. The number at the end of the BP indicates the number of DEG involved in the pathway.

Liquid chromatography–tandem mass spectrometry (LC–MS/MS) was used to identify proteins in conceptus and endometrial mono‐culture and co‐culture media. Based on PCA and Pearson correlation heatmap, the Control Med (base media) proteomic environment was clearly influenced by tissue culture, particularly when culture involved endometrium (Figure 4A,B). Although differences between IVD and IVP Conc mono‐culture media proteins were modest as evidenced by PCA, when IVD or IVP conceptus tissue were co‐cultured with endometrium (Endo‐IVD Conc or Endo‐IVP Conc), this resulted in two very distinct proteomic environments dependent on conceptus type and different from that of the endometrium alone (Endo; Figure 4A). Notably, compared to Endo, the IVP conceptus‐induced shift in the proteomic environment appeared more modest compared to that induced by the IVD conceptus (Figure 4A).

Compared to IVD Conc media, the IVP Conc media had 330 more‐ and 229 less abundant differentially abundant proteins (DAP). Four BP (Figure 4C) and one KEGG pathway related to translation, protein stabilization and metabolism were associated with the more abundant IVP Conc DAP (FDR ≤ 0.05). Compared to Control Med, IVD Conc and IVP Conc media contained 1108 and 1048 DAP, respectively (*p* < 0.05). When DAP were compared using a Venn diagram, 733 (*n* = 672 more‐ and 61 less abundant) DAP were identified as shared between the IVD and IVP conceptus mono‐cultures (Figure 4D). Thirty‐two BP and thirty‐three KEGG pathways were associated with the shared more abundant DAP of which the most significant BP and KEGG pathway was translation (FDR < 0.001; Figure 4E) and the ribosome (FDR < 0.001), respectively. Proteostasis was highly represented although the glycolytic process and TCA cycle (FDR < 0.05) were also identified BP. Three shared proteins including IFNT, sphingomyelin phosphodiesterase acid‐like 3A (SMPDL3A), and ribosomal protein lateral stalk subunit P0 (RPLP0) were a top 10 more abundant protein (LogFC > 23) in both the IVD and IVP Conc media. Folate receptor alpha (FOLR1) was the most abundant protein in the IVD Conc media but was the thirty‐fourth most abundant in the IVP Conc media. No BP were associated with the more abundant IVD (*n* = 217) or IVP (*n* = 249) Conc specific DAP, however, one KEGG pathway (metabolic pathways) was associated with the former (FDR < 0.05) and four KEGG pathways were associated with the latter including carbon metabolism, metabolic pathways, Parkinson's disease, and biosynthesis of amino acids (FDR < 0.05).

### 3.4 Co‐Culture IVD or IVP Conceptus‐Endometrium Media Protein

Compared to Control Med, Endo, Endo‐IVD Conc, and Endo‐IVP Conc media contained 3046, 2832, and 2875 DAP, respectively (*p* < 0.05). Compared to Endo‐IVD Conc media, Endo‐IVP Conc media contained 499 more‐ and 270 less abundant DAP. Three BP were associated with the more abundant DAP including, from most to least significant, (1) protein transport, (2) positive regulation of cell growth, and (3) intracellular protein transport (*p* < 0.05; Figure 4F). Four KEGG pathways were also identified including, from most to least significant, (1) proteoglycans in cancer, (2) platelet activation, (3) regulation of actin cytoskeleton, and (4) Rap1 signaling pathway (FDR < 0.05). No BP were associated with less abundant proteins, however, two KEGG pathways were identified including metabolic pathways and complement and coagulation cascades (*p* < 0.05).

The Endo‐IVD Conc or Endo‐IVP Conc media were then compared to Endo media to identify proteins dependent on co‐culture. Analysis resulted in identification of 1031 (*n* = 288 more‐ and 743 less abundant) and 604 (*n* = 195 more‐ and 409 less abundant) DAP in the Endo‐IVD Conc and Endo‐IVP Conc media, respectively (*p* < 0.05), indicating the Endo‐IVP Conc proteomic environment was less diverse from that of Endo as represented in the PCA plot (Figure 4A). Using the DAP, a Venn diagram was plotted to identify co‐culture proteins shared or specific to conceptus type (Figure 4G). Of the shared more abundant DAP (*n* = 123), no significant BP or KEGG pathways were identified, however, 15 KEGG pathways were associated with the shared less abundant DAP (*n* = 246) of which the complement and coagulation cascade was the most significant (FDR < 0.01). Regarding the more abundant proteins specific to the Endo‐IVD Conc media (*n* = 162), no significant BP or KEGG pathways were identified. Three BP and 20 KEGG pathways were associated with the less abundant DAP (*n* = 497) of which protein transport (Figure 4H) and Huntington's disease was the most significant BP and KEGG pathway (FDR < 0.001), respectively. Similarly, no BP or KEGG pathways were associated with the more abundant Endo‐IVP Conc specific DAP (*n* = 72). The three most abundant proteins specific to the Endo‐IVP Conc media, from greatest to least, included (1) aspartoacylase (ASPA), (2) melanotransferrin (MELTF), and (3) an unidentified protein (O46773) related to MHC class I heavy chain. Only one KEGG pathway (complement and coagulation cascade; FDR < 0.05) was associated with less abundant Endo‐IVP Conc specific DAP (163). Of note, five less abundant proteins specific to Endo‐IVP Conc media were associated with in utero embryonic development, however, this BP was not significant (*p* = 0.009; FDR = 1.00). These proteins included a fibroblast growth factor receptor (FGFR1), adducin 1 (ADD1), receptor protein‐tyrosine kinase (TIE1), myosin‐9 (MYH9), and drebin 1 (DBN1). Identified proteins in conceptus and endometrial mono‐culture and co‐culture media and associated comparisons are presented in Data S4. Identified proteins commonly localized to the extracellular environment (secreted) as well as conceptus and endometrial transcripts encoding proteins commonly localized to the plasma membrane (possible receptors) are presented in Data S5.

## 4 Discussion

This study was designed to evaluate the IVP bovine conceptus microenvironment during conceptus elongation, when crosstalk is enhanced between the conceptus and endometrium. Major findings include (1) the IVP conceptus has an altered biochemical response to the endometrium in the form of a reduced number of transcribed genes, mRNA abundance for genes transcribed, and increased expression of pro‐inflammatory mediators, (2) the IVP conceptus induced transcripts in the endometrium reflect an abnormal inflammatory response to pregnancy, and (3) the IVP conceptus elicits a suboptimal protein microenvironment with the endometrium that may reflect subsequent pregnancy failure.

During the first 3 weeks of gestation, endometrial secretions produced by the luminal and glandular epithelium completely support bovine conceptus elongation and growth. Referred to as histotroph, secreted products include various proteins, metabolites, amino acids, and other small molecules as well as extracellular vesicles, stimulating trophoblast proliferation, migration and secretion which involves modification of the conceptus transcriptome. Studies suggest embryonic DNA methylation and gene transcription are altered by in vitro culture systems [8, 10, 38, 39]. Specifically, cleaved bovine blastocysts collected from the reproductive tract and cultured in vitro until blastocyst formation had a greater number of hypermethylated loci and reduced transcription compared to IVD controls [10]. This would explain the discrepancy in the IVP and IVD conceptus transcriptomes in response to the endometrium in this study, as IVP conceptuses had a reduced number of DEG (*n* = 3356 and 1430, Endo‐IVD Conc and Endo‐IVP Conc, respectively) and reduced LogFC in DEG expression compared to the mono‐cultured tissue. Further, 90 DEG upregulated in IVD conceptus but not IVP conceptus tissue were associated with mRNA translation or ribosomal subunit biogenesis indicating IVP conceptuses expressed fewer genes supportive of protein synthesis. A direct comparison between co‐cultured IVD and IVP conceptus tissue and subsequent phenotype analysis identified over 80 DEG in IVP conceptuses associated with abnormal embryonic or fetal development. These included important developmental controls genes such as cell division cycle 20 (*CDC20*) and *SLC40A1*, the latter of which was downregulated in IVP conceptus tissue.

Solute carrier 40A1 or ferroportin is a plasma membrane iron exporter that maintains cell iron and reactive oxygen species (ROS) homeostasis. It is the only known iron exporter in mammals and degradation of ferroportin results in intracellular accumulation of iron, ROS and cell death, processes that occur during ferroptosis [40]. Prolonged iron supplementation to early murine embryos was found to decrease blastocyst formation and in vitro embryo culture contributes to murine and bovine embryo ROS accumulation [41, 42, 43]. Of relevance, the protein melanotransferrin (MELTF), an iron binding molecule that increases cellular uptake of iron [44], was also abundant in IVP conceptus co‐culture media. Further, two hemoglobin genes, hemoglobin A (*HBA*) and mu (*HBM*), were not expressed in IVP conceptus tissue but were the two most abundant IVD conceptus specific DEG. Hemoglobin A and HBM may support trophoblast oxygen tension and oxidative phosphorylation during elongation, but they may also protect the conceptus from ROS by acting as free radical scavengers [22, 45]. It was recently reported that HBA subunit alpha 1 functions as mitochondria‐associated antioxidant in T‐lymphocytes that when disrupted in rodents, results in exacerbated accumulation of ROS and inflammatory molecule expression [45]. Collectively, the IVP conceptus trophectoderm may be susceptible to intracellular iron accumulation, ROS, and ferroptosis, through abnormal expression of ferroportin and conceptus hemoglobin.

Although endometrial induced changes to the IVP conceptus transcriptome were modest compared to their IVD counterparts, the endometrium responded to IVP conceptus tissue with a larger number of DEG (*n* = 2700 and 3529, Endo‐IVD Conc and Endo‐IVP Conc, respectively). Inflammatory response was a major BP associated with the 663 more abundantly expressed endometrial DEG specific to IVP conceptuses. These included several genes involved in innate immune cell chemotaxis (*CCL1*, −*5*, −*11*, −*26*, *CCR1*, and *CXCL12*), T cell activation and proliferation [cluster of differentiation (*CD*)−*5*, −*28*, −*86*, −*247*, *CXCR6*, SH2 domain containing 2A (*SH2D2A*), and IL‐2 T cell kinase (*ITK*)] and interferon gamma (IFNG) signaling (*IL‐23R*, *IL‐18*, *IL‐12RB* and *IFNGR1*) indicating endometrium cultured with IVP conceptus tissue reflected an inflammatory and autoimmune state. Endo‐IVP Conc specific DEG and co‐culture media proteins that may have contributed to the inflammatory activities include an uncharacterized MHC class I heavy chain molecule (UniProt: O46773) and *BLA‐DQB*.

Specifically, *BLA‐DQB* encodes a MHC‐Class II molecule that is normally expressed by antigen presenting immune cells (macrophages, dendritic and B cells) to provide pathogen derived antigens to CD4^+^ T cells, triggering T cell proliferation and a cell mediated immune response. Bovine trophoblast cells reportedly lack MHC‐Class II and have reduced expression of MHC‐Class I molecules during the first trimester of pregnancy, a mechanism proposed to help the conceptus avoid maternal immune recognition [46]. It is possible that expression of MHC‐Class II within IVP conceptus tissue could have led to endometrial T cell recognition of conceptus antigens, contributing to an increase in inflammatory related DEG and processes within the endometrium. During a study investigating pregnancy failure associated with SCNT derived bovine embryos, Day 35 trophectoderm from SCNT derived conceptuses had elevated expression of MHC‐Class I molecules compared to IVD controls (AI generated) which was associated with immune cell infiltration into the endometrium [46]. Considering our study involved short‐term ex vivo tissue culture, the immune related endometrial DEG identified in this study must be in resident immune cells.

A lack of IVP conceptus expression of immunosuppressive molecules may have also contributed to the endometrial inflammatory environment. Of note, an MHC Class Ib molecule (non‐classical; E1BI37; BoLA) that may provide immunosuppression to pregnancy similar to human leukocyte antigen (HLA)‐G and HLA‐E, was detected within the Endo‐IVD Conc co‐culture media but not media associated with IVP conceptuses (mono‐ or co‐culture) [22]. Expression of galectin‐1 (*LGALS1*) was also less in Endo‐IVP Conc compared to Endo‐IVD Conc tissue. Galectins interact with N‐linked glycosylated proteins, including cluster of differentiation (CD) molecules, to modulate leukocyte activities. Research in rodents and cattle suggest that *LGALS1* provides immunotolerance to pregnancy through mediation of the endometrial Th1‐Th2 shift and increased regulatory T cell activity (Treg) activity [30, 47]. Galectin‐1 also dampens chronic inflammation by inducing apoptosis of Th17 lymphocytes that express IL‐17, which was more abundant in endometrium co‐cultured with IVP conceptus tissue [48]. Further, N‐linked glycosylation was a BP associated with less abundantly expressed endometrial DEG in response to IVP conceptus tissue which would severely impact endometrial protein function and immune cell activities. Indeed, alterations to N‐linked glycosylation results in rampant tissue inflammation and autoimmunity [49].

Interestingly, IVD but not IVP conceptuses decreased expression of several genes in endometrium associated with mitochondria function [ATP membrane subunit F (*ATP5F*), NADH:ubiquinone oxidoreductase (*NDUF*), and mitochondria RNA processing (*MRP*) subunits], the tricarboxylic acid (TCA) cycle [malate dehydrogenase 1 (*MDH1*), isocitrate dehydrogenase 2 (*IDH2*), and pyruvate dehydrogenase E1 subunit beta (*PDHB*)] and control of ROS accumulation particularly involving glutathione [superoxide dismutase 1 (*SOD1*), peroxiredoxin‐2 (*PRDX2*), −3, and −5, glutathione reductase (*GSR*), thioredoxin reductase 2 (*TXNRD2*), glutathione S‐transferase pi 1 (*GSTP1*) and glutathione peroxidase 4 (*GPX4*)]. Decreased cell mitochondria activity and decreased expression of genes related to ROS homeostasis can lead to elevated tissue oxygen concentrations and ROS accumulation [50]. It is possible that IVD conceptuses decrease endometrial mitochondria activity to increase trophoblast oxygen availability [22]. As indicated, *HBA* and *HBM* were the two most up‐regulated DEG specific to Endo‐IVD Conc tissue and several of the mitochondria and ROS homeostatic genes that decreased in expression in endometrium during Endo‐IVP Conc co‐cultures were exclusively up‐regulated in the Endo‐IVD Conc tissue (not up‐regulated in the Endo‐IVP Conc). These included *NDUF* and *MRP* subunits as well as *IDH2*, *PRDX2*, −*4*, −*5*, *TXNRD2*, *GSTP1*, and *GPX4*. Theoretically, up‐regulation of enzymes important for ROS homeostasis in the conceptus could protect the trophoblast from endometrial or self‐derived ROS to support trophoblast function. Accumulation of ROS in BeWo cells, a human choriocarcinoma cell line, resulted in a 5‐fold reduction in BeWo fusion and reduced expression of placental hormones including placental lactogen (PL) [51]. During early pregnancy in cattle, giant binucleate trophoblast cells will fuse with the maternal epithelium during establishment of the syneptheliochorial placenta and secrete several placental hormones including PL [52, 53].

Regarding the endometrium, short term conceptus induced hypoxia could also support early pregnancy by modulating the maternal immune response. Evidence suggests that short term tissue hypoxia and ROS accumulation promotes immune suppression and extracellular vesicle secretion, endometrial processes that are important during early pregnancy in mammals [54, 55, 56, 57]. Although macrophages and neutrophils will use ROS to promote parasite killing via the “oxidative burst”, it was also found that some parasites will trigger host tissue mitochondria dysfunction and ROS accumulation to suppress the host immune response and promote parasite growth within tissues [58, 59, 60]. This is a recent finding and perhaps bovine trophoblast use a similar mechanism, inducing short term mitochondria dysfunction and ROS accumulation within local endometrium to dampen the maternal immune response. Theoretically, a simultaneous increase in molecules that promote ROS homeostasis within the trophoblast would protect the early placenta during this process, a mechanism also used by some parasites [61]. However, we did not measure tissue ROS in this study and further testing is needed to validate this hypothesis.

Although IVP conceptus tissue induced a greater transcriptional response in the endometrium in terms of number of DEG, the proteomic environment associated with the Endo‐IVP Conc co‐culture media was considerably less diverse compared to the Endo‐IVD Conc media (*n* = 1031 and 604 DAP, Endo‐IVD Conc and Endo‐IVP Conc, respectively). Several BP associated with less abundantly expressed DEG in endometrium co‐cultured with IVP conceptus tissue (Endo‐IVP Conc) were related to protein processing or transport within the cytosol suggesting reduced proteostasis and secretory capacity of the endometrium. This is evidenced in the proteomic PCA plot presented in Figure 4 as the Endo‐IVP Conc media samples were more similar to the mono‐cultured endometrium. Various important metabolic proteins such as prostaglandin D2 synthase (PTGDS), microsomal glutathione S‐transferase 1 (MGST1) and phosphofructokinase, platelet (PfKP) were less abundant in the Endo‐IVP Conc media compared to the Endo‐IVD Conc treatment. Proteins that were more abundant included proteases matrix metallopeptidase 2 (MMP2) and cathepsin L (CTSL) as well as proteoglycan syndecan‐1 (SDC1). When aberrantly expressed, these molecules are well‐known to be associated with abnormal tissue reorganization and cancer metastasis [62, 63]. The consequence of elevated protease abundance within the IVP conceptus‐endometrial microenvironment are unknown, particularly within the intercaruncular areas of endometrium where trophoblast attachment and tissue reorganization are minimal relative to the caruncular endometrium.

## 5 Conclusion

The elongating IVP bovine conceptus has an altered transcriptome and proteome driven largely by interactions with the endometrium that results in an abnormal endometrial response to pregnancy independent of maternal recognition. This includes an abnormal inflammatory state within the endometrium and suboptimal protein microenvironment surrounding the conceptus that if not overcome, likely results in early embryonic mortality and pregnancy failure. Thus, it seems that the endometrium “challenges” and subsequently “senses” conceptus quality, a difficult trial for the IVP conceptus. Methods that improve IVP embryo biochemistry before and after transfer, such as optimized embryo culture media, could reduce pregnancy failure associated with IVP embryo technologies. Alternatively, with identification of specific signaling molecules and pathways, it seems possible that immunotherapies or methods that control the endometrial response to pregnancy could mitigate pregnancy failure associated with the IVP conceptus.

## Author Contributions

**Katheryn D. Peterson:** animal work, data collection and organization, and manuscript preparation. **Trevor F. Freeman, Shankar P. Poudel, Susanta K. Behura, David Kakhniashvili, Daniel L. Johnson, Jonathan E. Beever, Elizabeth A. Shepherd and Thomas E. Spencer:** data collection, analysis and or interpretation. **Mary A. Oliver, Rebecca R. Payton, J. Lannett Edwards:** in vitro embryo production. **Tulio M. Prado and Lew G. Strickland:** animal work. **Daniel J. Mathew:** acquisition of funding, experimental design, animal work, data organization, and manuscript preparation.

## Conflicts of Interest

The authors declare no conflicts of interest.

## Supporting information


**Data S1:** fsb270951‐sup‐0001‐DataS1.xlsx.

**Data S2:** fsb270951‐sup‐0002‐DataS2.xlsx.

**Data S3:** fsb270951‐sup‐0003‐DataS3.xlsx.

**Data S4:** fsb270951‐sup‐0004‐DataS4.xlsx.

**Data S5:** fsb270951‐sup‐0005‐DataS5.xlsx.

**Data S6:** fsb270951‐sup‐0006‐DataS6.xlsx.

**Figure S1:** Uterine flush fluid (UFF) media protein identified by liquid chromatography–tandem mass spectrometry (LC–MS/MS). (A, B) Principal component analysis (PCA; A) and Pearson correlation heat map (B) of Day 16 cyclic heifer UFF (Endo/Cyclic UFF; *n* = 4) and UFF from heifers carrying Day 16 IVD (IVD/AI UFF; *n* = 4) or IVP (IVP/ET UFF; *n* = 5) conceptuses. (C) Venn diagram of Endo/Cyclic, IVD/AI and IVP/ET UFF DAP identified when compared to RPMI medium, the base medium used to flush uteri.

**Table S1:** Bovine GenBank accession number, gene name, primer direction, primer sequence, product size (base pair) and percent amplification efficiency (%E) of mono‐ and co‐culture endometrial cDNA amplified during real time quantitative‐polymerase chain reaction (RT‐qPCR).

**Table S2:** Mono‐ and co‐culture endometrium [Endo; (*n* = 13), Endo‐IVD Conc (*n* = 15) and Endo‐IVP Conc (*n* = 12)] real‐time quantitative polymerase chain reaction (RT‐qPCR) relative expression of target genes. Relative expression data were normalized to the geometric mean of reference genes *RNF11* and *YWHAZ*. Data are presented as least squares mean ± standard error of the least squares means (LSM ± SEM). Different letters indicate significant differences between treatments whereas *p* value corresponds to the overall effect of treatment. NS, non‐significant.

## Data Availability

RNA‐seq fastq and featureCount data are available within the national center for biotechnology information (NCBI) Gene Expression Omnibus (GEO; accession numbers: GSE256172 and GSE295732). All endometrial and conceptus tissue, RNA‐Seq differentially abundant gene and LC–MS/MS protein abundance data are available in Data [Link], [Link], [Link], [Link], [Link], [Link]. All other data associated with the study are fully available upon request from the corresponding author.
