# Supplementary material for: Bovine Endometrium Drives and Responds to Divergence of In Vitro Produced Conceptus Biochemistry
Source: FASEB J. 2025 Aug 19;39(16):e70951. doi: 10.1096/fj.202501962R (PMC12363384; doi:10.1096/fj.202501962R)
Supplement: Supplementary file 7 — Figure S1: Uterine flush fluid (UFF) media protein identified by liquid chromatography–tandem mass spectrometry (LC–MS/MS). (A, B) Principal component analysis (PCA; A) and Pearson correlation heat map (B) of Day 16 cyclic heifer UFF (Endo/Cyclic UFF; n = 4) and UFF from heifers carrying Day 16 IVD (IVD/AI UFF; n = 4) or IVP (IVP/ET UFF; n = 5) conceptuses. (C) Venn diagram of Endo/Cyclic, IVD/AI and IVP/ET UFF DAP identified when compared to RPMI medium, the base medium used to flush uteri. [file FSB2-39-e70951-s006.docx]

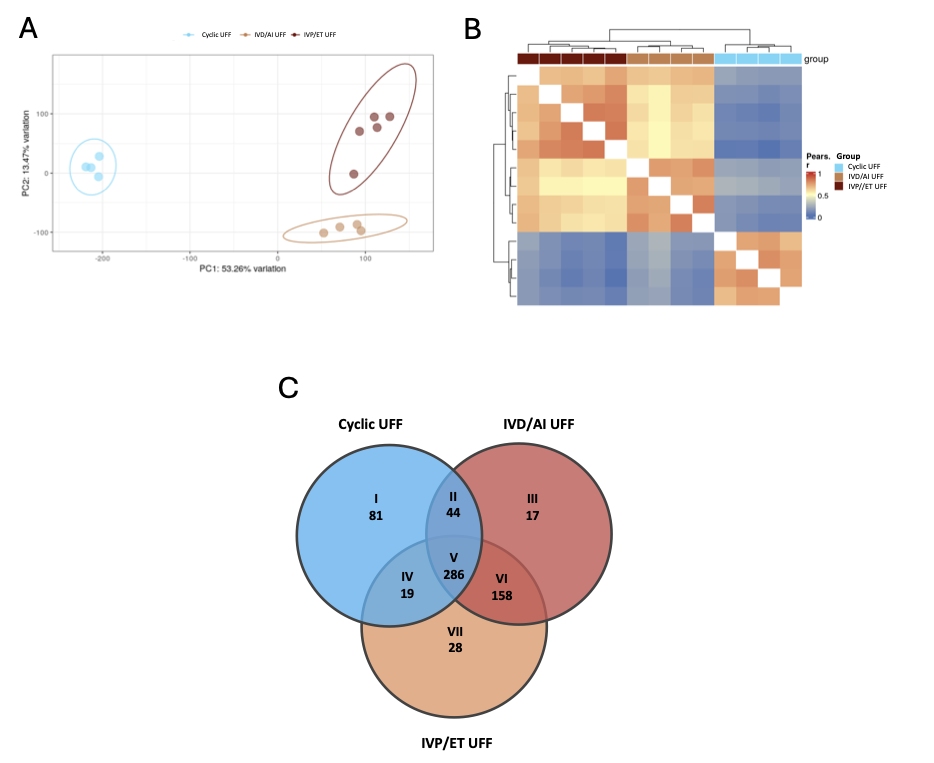
Supplemental Figure 1

Supplemental Figure 1. Uterine flush fluid (UFF) media protein identified by liquid chromatography-tandem mass spectrometry (LC-MS/MS). (A-B) Principal component analysis (PCA; A) and Pearson correlation heat map (B) of Day 16 cyclic heifer UFF (Endo/Cyclic UFF; n=4) and UFF from heifers carrying Day 16 IVD (IVD/AI UFF; n=4) or IVP (IVP/ET UFF; n=5) conceptuses. (C) Venn diagram of Endo/Cyclic, IVD/AI and IVP/ET UFF DAP identified when compared to RPMI medium, the base medium used to flush uteri.
