# Supplementary material for: Bovine Endometrium Drives and Responds to Divergence of In Vitro Produced Conceptus Biochemistry
Source: FASEB J. 2025 Aug 19;39(16):e70951. doi: 10.1096/fj.202501962R (PMC12363384; doi:10.1096/fj.202501962R)
Supplement: Supplementary file 8 — Table S1: Bovine GenBank accession number, gene name, primer direction, primer sequence, product size (base pair) and percent amplification efficiency (%E) of mono‐ and co‐culture endometrial cDNA amplified during real time quantitative‐polymerase chain reaction (RT‐qPCR). [file FSB2-39-e70951-s002.docx]

| **GenBank Accession** | **Gene** | **Primer** | **Primer Sequence (3’-5’)** | **Product Size** | **%E** |
| --- | --- | --- | --- | --- | --- |
|  |  |  |  |  |  |
| XM_024995021.1 | ***CD80*** | Forward | ATCCCAGTCTCAGCATTTGG | 87 | 110.99 |
|  |  | Reverse | CTGTCTGCGTATTGCAGCAT |  |  |
| NM_174301.3 | ***CXCR4*** | Forward | AAGGCTCAGAAGCGCAAG | 98 | 102.00 |
|  |  | Reverse | GAGTCGATGCTGATCCCA |  |  |
| NM_001015511.4 | ***IFNT*** | Forward | TGCAGGACAGAAAAGACTTTGGT | 69 | 113.01 |
|  |  | Reverse | CCTGATCCTTCTGGAGCTGG |  |  |
| NM_174093.1 | ***IL1B*** | Forward | ACCTGAACCCATCAACGAAATG | 74 | 100.92 |
|  |  | Reverse | TAGGGCCATCAGCCTCAAATAACA |  |  |
| NM_173923.2 | ***IL6*** | Forward | CGCATGGTCGACAAAATCTCT | 69 | 102.21 |
|  |  | Reverse | GCTGCTTTCACACTCATCATTCTT |  |  |
| [XM_005215801.4](https://www.ncbi.nlm.nih.gov/entrez/viewer.fcgi?db=nucleotide&id=1387208692) | ***IL18*** | Forward | TCTTTGAGGATATGCCTGATTCTG | 95 | 105.94 |
|  |  | Reverse | CAGACCTCTAGTGAGGCTGTCCTT |  |  |
| NM_174366.1 | ***ISG15*** | Forward | CCAACCAGTGTCTGCAGAGA | 76 | 97.24 |
|  |  | Reverse | CCCTAGCATCTTCACCGTCA |  |  |
| NM_001015570.3 | ***LGALS9*** | Forward | TCAGCTTCCAGCCTCCAGGG | 86 | 96.00 |
|  |  | Reverse | TCCAGGGGCGCTGTGTATGGT |  |  |
| NM_173940.2 | ***MX1*** | Forward | CGAGCCGAGTTCTCCAAATG | 114 | 96.00 |
|  |  | Reverse | CAACTCTCTGCCACGATACC |  |  |
| NM_001040606.1 | ***OAS1*** | Forward | CCCGGCGGACCCTACAGGAA | 84 | 94.62 |
|  |  | Reverse | TCCAGCCAGACCAAAGCCGC |  |  |
| [NM_001081577.1](https://www.ncbi.nlm.nih.gov/entrez/viewer.fcgi?db=nucleotide&id=126158902) | ***SLC1A4*** | Forward | CGGTCCCCAAAGAGACAG | 128 | 105.60 |
|  |  | Reverse | AGCGCTCGTGTTGTAGGTC |  |  |
| XM_024984913.1 | ***SLC6A9*** | Forward | CCAGTACCAGCCAATCACCT | 82 | 99.73 |
|  |  | Reverse | AGAGGACAGAGCCATGAGGA |  |  |
| XM_005212025.3 | ***SLC39A9*** | Forward | GGAGGTGTTTGCCTGTTGAT | 132 | 106.00 |
|  |  | Reverse | TCACCACCAGCAGTAAGCAG |  |  |
| NM_001077953.1 | ***RNF11*** | Forward | TCCGGGAGTGTGTGATCTGTATGAT | 131 | -- |
|  |  | Reverse | GCAGGAGGGGCACGTGAAGG |  |  |
| NM_174313 | ***YWHAZ*** | Forward | ACCTACTCCGGACACAGAACATC | 65 | -- |
|  |  | Reverse | CCAGTTTGGCCTTCTGTACCA |  |  |

Supplemental Table 1. Bovine GenBank accession number, gene name, primer direction, primer sequence, product size (base pair) and percent amplification efficiency (%E) of mono- and co-culture endometrial cDNA amplified during real time quantitative-polymerase chain reaction (RT-qPCR).
