# Supplementary material for: Bovine Endometrium Drives and Responds to Divergence of In Vitro Produced Conceptus Biochemistry
Source: FASEB J. 2025 Aug 19;39(16):e70951. doi: 10.1096/fj.202501962R (PMC12363384; doi:10.1096/fj.202501962R)
Supplement: Supplementary file 9 — Table S2: Mono‐ and co‐culture endometrium [Endo; (n = 13), Endo‐IVD Conc (n = 15) and Endo‐IVP Conc (n = 12)] real‐time quantitative polymerase chain reaction (RT‐qPCR) relative expression of target genes. Relative expression data were normalized to the geometric mean of reference genes RNF11 and YWHAZ. Data are presented as least squares mean ± standard error of the least squares means (LSM ± SEM). Different letters indicate significant differences between treatments whereas p value corresponds to the overall effect of treatment. NS, non‐significant. [file FSB2-39-e70951-s003.docx]

| **Gene** | **Endo** | **Endo-IVD Conc** | **Endo-IVP Conc** | **P value** |
| --- | --- | --- | --- | --- |
|  |  |  |  |  |
| *CD80* | 0.88 ± 0.11^a^ | 1.14 ± 0.1 ^a^ | 1.25 ± 0.12 ^a^ | NS |
| *CXCR4* | 1.40 ± 0.15 ^a^ | 0.91 ± 0.14 ^a^ | 0.98 ± 0.15 ^a^ | NS |
| *IFNT* | 1.08 ± 0.18 ^a^ | 1.10 ± 0.17 ^a^ | 1.26 ± 0.19 ^a^ | NS |
| *IL18* | 0.84 ± 0.18 ^a^ | 1.14 ± 0.16 ^a^ | 1.41 ± 0.18 ^a^ | NS |
| *IL1B* | 2.69 ± 0.51 ^a^ | 0.91 ± 0.48 ^a^ | 1.05 ± 0.56 ^a^ | NS |
| *IL6* | 1.40 ± 0.30 ^a^ | 1.17± 0.28 ^a^ | 1.13 ± 0.33 ^a^ | NS |
| *ISG15* | 0.05 ± 0.39 ^a^ | 4.86 ± 0.36 ^b^ | 6.87 ± 0.42^c^ | < 0.001 |
| *LGALS9* | 0.39 ± 0.15 ^a^ | 1.88 ± 0.14 ^b^ | 1.58 ± 0.16 ^b^ | < 0.001 |
| *MX1* | 0.11 ± 0.29 ^a^ | 3.39 ± 0.27 ^b^ | 3.55 ± 0.32 ^b^ | < 0.001 |
| *OAS1* | 0.09 ± 0.25 ^a^ | 3.67 ± 0.24 ^b^ | 3.57 ± 0.27 ^b^ | < 0.001 |
| *SLC1A4* | 1.10 ± 0.17 ^a^ | 1.11 ± 0.16 ^a^ | 1.11 ± 0.19 ^a^ | NS |
| *SLC39A9* | 1.30 ± 0.15 ^a^ | 1.07 ± 0.14 ^a^ | 0.93 ± 0.16 ^a^ | NS |
| *SLC6A9* | 0.75 ± 0.11 ^a^ | 1.27 ± 0.10^b^ | 1.12 ± 0.12 ^b^ | < 0.05 |

Supplemental Table 2. Mono- and co-culture endometrium [Endo; n=13), Endo-IVD Conc (n=15) and Endo-IVP Conc (n=12)] real-time quantitative polymerase chain reaction (RT-qPCR) relative expression of target genes. Relative expression data were normalized to the geometric mean of reference genes *RNF11* and *YWHAZ*. Data are presented as least squares mean ± standard error of the least squares means (LSM ± SEM). Different letters indicate significant differences between treatments whereas P value corresponds to the overall effect of treatment. NS, non-significant.
